# Correction: Rapid progression of mitral‑aortic intervalvular fibrosa pseudoaneurysm in a patient with bicuspid aortic valve endocarditis: a case report and comprehensive literature review

**DOI:** 10.1007/s40477-026-01173-5

**Published:** 2026-06-23

**Authors:** Lanhua Chen, Rong Chen, Yunhai Liao, Huizhong Li

**Affiliations:** 1 Department of Ultrasound Diagnosis, 900th Hospital of Joint Logistics Support Force of PLA, Fuzhou, Fujian China; 2 Department of Emergency, The Second Affiliated Hospital of Fujian University of Traditional Chinese Medicine, Fuzhou, Fujian China

**Correction: Journal of Ultrasound** **https://doi.org/10.1007/s40477-026-01160-w**

The order of authors has been incorrectly published in the original publication. The complete correct order of authors is given below.

Lanhua Chen^1^, Rong Chen^1^, Yunhai Liao^2^, Huizhong Li^1^

The original article has been corrected.

